# Is the Prevalence of Overactive Bladder Overestimated? A Population-Based Study in Finland

**DOI:** 10.1371/journal.pone.0000195

**Published:** 2007-02-07

**Authors:** Kari A.O. Tikkinen, Teuvo L.J. Tammela, Aila M. Rissanen, Antti Valpas, Heini Huhtala, Anssi Auvinen

**Affiliations:** 1 Department of Urology, Tampere University Hospital, Tampere, Finland; 2 Medical School, University of Tampere, Tampere, Finland; 3 Tampere School of Public Health, University of Tampere, Tampere, Finland; 4 Obesity Research Unit, Helsinki University Central Hospital, Helsinki, Finland; 5 Department of Obstetrics and Gynecology, South Karelian Central Hospital, Lappeenranta, Finland; Ludwig Boltzmann Institute for Urological Oncology, Austria

## Abstract

**Background:**

In earlier studies, one in six adults had overactive bladder which may impair quality of life. However, earlier studies have either not been population-based or have suffered from methodological limitations. Our aim was to assess the prevalence of overactive bladder symptoms, based on a representative study population and using consistent definitions and exclusions.

**Methodology/Principal Findings:**

The aim of the study was to assess the age-standardized prevalence of overactive bladder defined as urinary urgency, with or without urgency incontinence, usually with urinary frequency and nocturia in the absence of urinary tract infection or other obvious pathology. In 2003–2004, a questionnaire was mailed to 6,000 randomly selected Finns aged 18–79 years who were identified from the Finnish Population Register Centre. Information on voiding symptoms was collected using the validated Danish Prostatic Symptom Score, with additional frequency and nocturia questions. Corrected prevalence was calculated with adjustment for selection bias due to non-response. The questionnaire also elicited co-morbidity and socio-demographic information. Of the 6,000 subjects, 62.4% participated. The prevalence of overactive bladder was 6.5% (95% CI, 5.5% to 7.6%) for men and 9.3% (CI, 7.9% to 10.6%) for women. Exclusion of men with benign prostatic hyperplasia reduced prevalence among men by approximately one percentage point (to 5.6% [CI, 4.5% to 6.6%]). Among subjects with overactive bladder, urgency incontinence, frequency, and nocturia were reported by 11%, 23%, and 56% of men and 27%, 38%, and 40% of women, respectively. However, only 31% of men and 35% of women with frequency, and 31% of subjects of both sexes with nocturia reported overactive bladder.

**Conclusions/Significance:**

Our results indicate a prevalence of overactive bladder as low as 8% suggesting that, in previous studies, occurrence has been overestimated due to vague criteria and selected study populations regarding age distribution and low participation.

## Introduction

Research on urinary storage problems has focused on incontinence in women, but during recent years other urinary storage problems (urgency, frequency, and nocturia) and their treatment among both sexes has commanded attention worldwide [1]. According to the International Continence Society, overactive bladder is a symptom-defined condition characterized by urinary urgency, with or without urgency incontinence, usually with urinary frequency and nocturia. The term overactive bladder is appropriate if there is no proven infection or other obvious pathology [2].

Overactive bladder is a poorly understood disorder [1]. In earlier reports, overactive bladder impaired quality of life [3], [4], was underdiagnosed and undertreated [3], [5]–[10], and cost more than $9 billion in the United States in 2000 [4], [11]. However, the value of current overactive bladder treatment with antimuscarinics (with an increasing market [11% annual growth] worldwide of more than $2.2 billion in 2005 [12]) was questioned by the Cochrane Review [13].

Most earlier studies on overactive bladder have reported a prevalence of 10%–20% [3]–[7], [9], [14]–[16], the most widely cited studies estimating prevalence of overactive bladder as one in six [3], [4]. Some studies have reported prevalence as high as 30% to 53% [8], [10], while one showed only 8% [17], and one as low as 2% [18]. Unfortunately, all these studies have had methodological limitations [3], [4], [6], [7], [9], [14]–[17] or have not been population-based [5], [8], [10], [18].

We assessed the prevalence of overactive bladder in a population-based study of subjects of both sexes aged 18–79 years.

## Methods

### Study design

Between November 2003 and February 2004, a questionnaire was mailed to a random sample of 3,000 men and 3,000 women aged 18–79 years drawn from the Finnish Population Register Center. Stratification by age was used in subject selection, with oversampling of the younger age groups to achieve a similar number of subjects with urgency/overactive bladder even in age groups with lower prevalence of urgency/overactive bladder (Table S1). We selected the target level of accuracy so that, given a true prevalence of 15%, we could exclude a prevalence of 10% or lower. Information on voiding symptoms was collected using the validated Danish Prostatic Symptom Score (Table 1) [19]. The questionnaire included items related to physician-diagnosed co-morbidity (such as gynecological, internal, mental, musculoskeletal, neurological, and/or urological conditions), prescribed medication (over the last 3 months) and socio-demographic factors (such as marital, educational and employment status). Information on pregnancy was based on both questionnaire and data from the Finnish Population Register Center which also provided information on puerperium and urbanity. Questionnaires were first mailed in late November 2003, with reminders a month later. To persons who did not respond, final questionnaire was sent in February 2004. The last questionnaires were returned in June 2004. In accordance with Finnish regulations on questionnaire surveys, an exemption from ethical review was granted by the ethical committee of Tampere University Hospital (Tampere, Finland).

**Table 1:** Overactive bladder symptom-related questions and definitions of the study in Finland, 2003–2004

Symptoms	Defining questions (with answer options)	Normal	Abnormal
Urgency and overactive bladder*	“Do you experience an imperative (strong) urge to urinate?” with answer options: never-rarely-often-always.	Never or rarely	Often or always
Urgency incontinence	“Is the urge so strong that urine starts to flow before you reach the toilet?” with answer options: never-rarely-often-always.	Never or rarely	Often or always
Frequency	“How many times did you usually urinate per day during the last month?”	≤8 voids per day	>8 voids per day
Nocturia	“How many times do you have to void per night?” and “How many times did you most typically get up to urinate from the time you went to bed at night until you got up in the morning?” were combined.	≤1 void per night	>1 void per night

* Urgency classification without any exclusions; overactive bladder classification was performed after exclusion of subjects with urinary tract infection or other obvious pathology (Table 2).

### Exclusions and definitions

**Figure 1:**
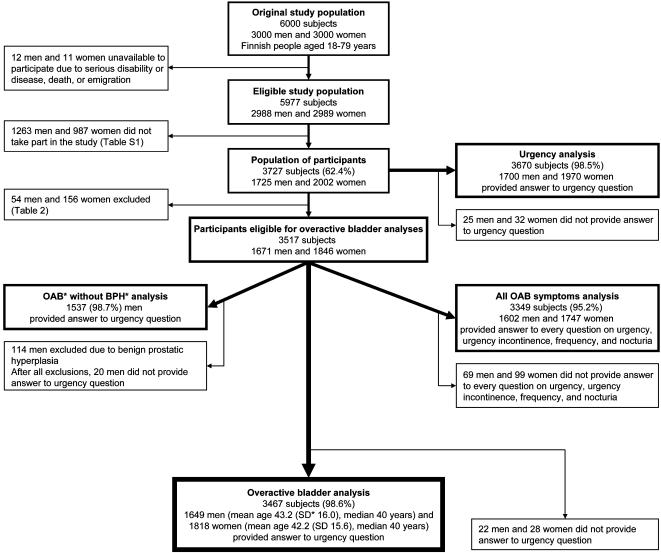
Flow chart of the study in Finland, 2003–2004. * OAB, overactive bladder; BPH, benign prostatic hyperplasia; SD, standard deviation.

In the first analysis (Urgency analysis), we assessed the prevalence of urgency in adult population without applying any exclusion criteria (Figure 1). In the main analysis (Overactive bladder analysis), we assessed the prevalence of overactive bladder in adult population excluding those with physician-diagnosed: 1) chronic or acute urinary tract infection (in the past 2 weeks); 2) genitourinary cancer (excluding renal); or 3) contracted bladder (due to radiation or painful bladder syndrome), also 4) prescribed loop diuretics; and 5) pregnant or puerperal women, with puerperium defined as 6 weeks after childbirth (Table 2). In the third analysis (OAB without BPH analysis), in addition to above mentioned exclusions (of Overactive bladder analysis), we excluded men reporting physician-diagnosed benign prostatic hyperplasia. We performed a fourth analysis (All OAB symptoms analysis), to assess the relationship of all symptoms of overactive bladder with the same exclusions as used in the Overactive bladder analysis (Figure 1).

**Table 2:** Exclusions of the study population of overactive bladder analysis: number of excluded subjects among 1725 men and 2002 women in Finland, 2003–2004

Characteristics	Men	Women	Both sexes
Urinary tract infection*	16	59	75
Genitourinary cancer†	22	11	33
Contracted bladder‡	2	5	7
Puerperium§		8	8
Pregnancy		49	49
Taking loop-diuretics	19	30	49
**Together**	**54**	**156**	**210**

* Acute (in past 2 weeks) or chronic urinary tract infection.

† Excluding renal cancer.

‡ Due to e.g. painful bladder syndrome or radiation.

§ Puerperium defined as 6 weeks after childbirth.

The urgency question from the validated Danish Prostatic Symptom Score was used to assess the prevalence of urgency and overactive bladder [19]. Overactive bladder with or without urgency incontinence classification was subdivided into overactive bladder cases based on the urinary urgency incontinence question of the Danish Prostatic Symptom Score. The mean daily number of voids was used for urinary frequency classification, whereas responses to nocturia questions from the Danish Prostatic Symptom Score and American Urological Association Symptom Index were combined [19], [20]. The Danish Prostatic Symptom Score questionnaire was applied for the past 2 weeks, while frequency question and nocturia question of American Urological Association Symptom Index pertained to the past month (Table 1).

### Statistical analysis

Subjects were stratified into 10-year age groups (18–29, 30–39, 40–49, 50–59, 60–69, and 70–79 years). The age-standardized prevalence was calculated using the general population of Finland (end of year 2003) by the Finnish Population Register Centre [21] (and the European standard population [22]). Binomial regression with identity link was used with presence of overactive bladder as outcome for extrapolation of age-specific prevalence of overactive bladder among people aged 80 years or more. All confidence intervals were likelihood-based. Confidence Interval Analysis 2.0.0 software (Trevor Bryant, University of Southampton, United Kingdom) was used for calculating age-standardized prevalences and confidence intervals (CI). Other analyses were performed using the Stata 9 (StataCorp LP, College Station, United States).

Adjustment for selection bias due to non-response was made for each symptom and combination of symptom (urgency, overactive bladder with or without urgency incontinence, frequency, and nocturia) after possible exclusions. First, prevalence of symptoms was calculated by mailing round (defined by date of questionnaire completion). As the prevalence of symptoms was lower in the subsequent than in the first round responses, the prevalence among non-participants (of the eligible study population) was assumed to be similar to the late responders, using formula:
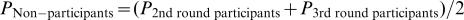
where *P* is prevalence of symptom (urgency, overactive bladder with or without urgency incontinence, frequency or nocturia).

Hence, based on the number of non-participants and prevalence of symptoms, we calculated the number of subjects with each symptom (and combinations in the All OAB symptoms analysis). The corrected prevalence of symptom was calculated using the formula:
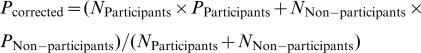
where *P* is prevalence of symptom (or combination of symptom) and *N* is number of subjects.

Concerning analyses for corrected prevalence of symptoms, we also performed an analysis excluding the same proportion of subjects among non-participants as we had done among participants, but the results did not materially change.

## Results

Of the 6,000 subjects approached for the study, 3,727 (62.4%) participated; 23 were unavailable because of serious disability or disease, death, or emigration (Figure 1). Of all participants, 98.5% (*n* = 3670) responded to the Danish Prostatic Symptom Score urgency question (Urgency analysis). For the assessment of overactive bladder prevalence (Overactive bladder analysis), we excluded 210 participants (Table 2). Most of the included participants (94.0%) also gave the date of questionnaire completion (Table S1). To assess the effect of benign prostatic hyperplasia on overactive bladder (OAB without BPH analysis), we further excluded 114 men. For comparison of all symptoms of overactive bladder, every question on urgency, urgency incontinence, frequency, and nocturia was answered by 95.2% of subjects (Figure 1).

**Figure 2:**
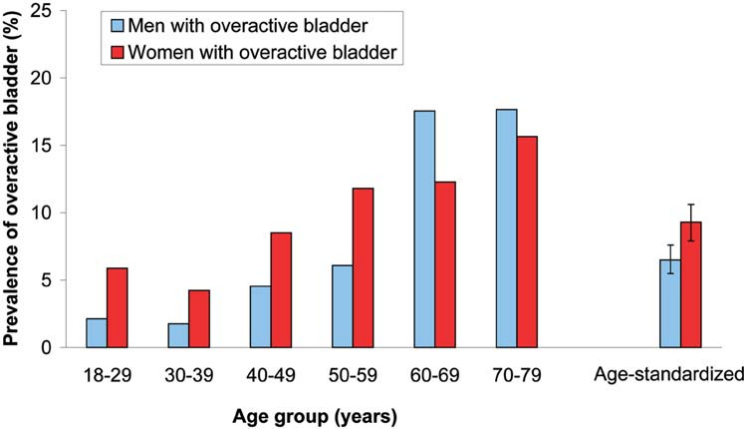
The prevalence of overactive bladder in Finland, 2003–2004. The blue bars indicate men with overactive bladder and the red bars women with overactive bladder. Age-standardization was performed using the general population [21].

After age-standardization (to the Finnish general population), prevalence of urgency was 7.0% (95% CI, 5.9% to 8.1%) for men and 10.3% (CI, 8.9% to 11.6%) for women. The age-standardised prevalence of overactive bladder was 6.5% (CI, 5.5% to 7.6%) for men and 9.3% (CI, 7.9% to 10.6%) for women (Figure 2). Exclusion of men with benign prostatic hyperplasia decreased the prevalence of overactive bladder among men to 5.6% (CI, 4.5% to 6.6%). The effect of benign prostatic hyperplasia on the prevalence of overactive bladder was strongest in elderly with the prevalence decreasing from 17.5% to 13.5% at age 60–69 years and from 17.6% to 14.0% at age 70–79 years after further exclusion of men with benign prostatic hyperplasia.

In general, overactive bladder was slightly more common among women than men after age-standardization (Figure 2). It was more common among women in younger ages while among men it was more common in those aged 60 years and above. Among men, the sharpest increase occurred at age 60–69 years while among women the increase was more steady. The mean increases in prevalence of overactive bladder were 2.4 percentage point (CI, 1.9%-point to 3.0%-point) per 10-year age group for men and 1.9 percentage point (CI, 1.2%-point to 2.6%-point) per 10 years for women. There was no statistically significant departure from linearity in either sex (Figure 2).

**Figure 3:**
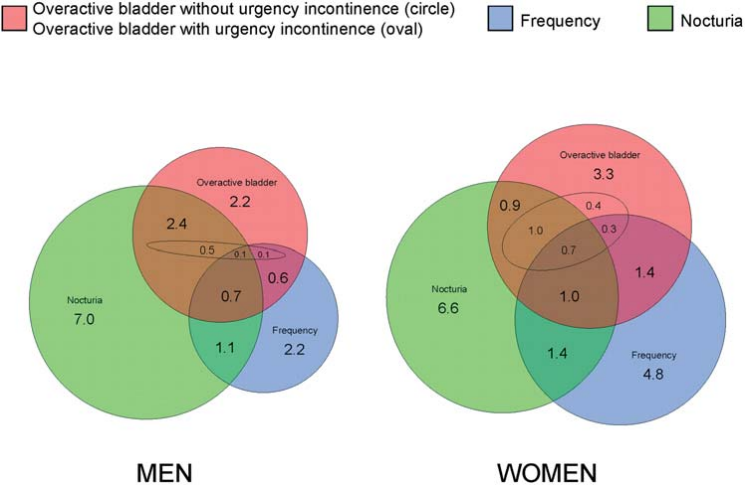
Age-standardized prevalence of overactive bladder symptoms among Finnish people aged 18–79 years, 2003–2004. The red circle represents subjects with overactive bladder without urgency incontinence excluding the area of the red oval representing subjects with overactive bladder with urgency incontinence. The blue circle represents subjects with urinary frequency (defined as more than eight voids per day) and the green circle nocturia (defined as more than one void per night). Age-standardization was performed using the general population [21].

In the All OAB symptoms analysis (Figure 3), urgency incontinence was reported by 11% of men and 27% of women among those with overactive bladder. Urinary frequency was reported by 23% of men and 38% of women with overactive bladder whereas the corresponding figures for nocturia were 56% and 40%. On the other hand, even though subjects with overactive bladder reported more frequency and nocturia than subjects without overactive bladder, only 31% of men and 35% of women with frequency, and 31% of subjects among both sexes with nocturia reported overactive bladder (Figure 3).

Without corrections for non-response, urgency was reported by 7.9% (CI, 6.5% to 9.3%) of men and 10.7% (CI, 9.0% to 12.3%) of women after age-standardization. The corresponding figures for overactive bladder were 7.3% (CI, 5.9% to 8.7%) for men and 9.7% (CI, 8.0% to 11.3%) for women. Further exclusion of men with benign prostatic hyperplasia decreased the non-corrected prevalence of overactive bladder to 6.3% (CI, 4.8% to 7.7%) among men aged 18–79 years.

## Discussion

In our study, the prevalence of overactive bladder was 6.5% for men and 9.3% for women, i.e. no more than 8% of adult population aged 18–79 years had overactive bladder. Subjects with overactive bladder reported more frequency and nocturia than those without overactive bladder, but the majority of subjects with frequency, or nocturia did not report overactive bladder.

The reported prevalence of overactive bladder has varied widely in earlier studies due to differences in symptom assessment, study population, data collection, and definition of overactive bladder including exclusion criteria. Most other studies have reported greater prevalence of overactive bladder than found in our study [3]–[10], [14]–[16]. Some [3]–[5], [15]–[17] but not all studies [6] have also reported more urgency incontinence among subjects with overactive bladder than we found.

The definition of a symptom-defined disorder, such as overactive bladder, has a major impact on outcome [23]. We used the overactive bladder definition of International Continence Society, with urgency (defined as sudden compelling desire to void) as a sufficient criterion for overactive bladder [2]. This definition is idealistic and ambiguous. The qualitative definition disregarding severity or symptom bother makes it difficult to apply. The classification of a symptom (including the time period during which the occurrence of symptoms is asked) strongly influences the result, due in part to fluctuating character and very high remission rates of lower urinary tract symptoms, including urgency [24]. We asked about urgency in the last 2 weeks with four response option: if urgency was reported “never” or “rarely”, the subject was classified as normal, while “often” and “always” were regarded as abnormal. Our classification of urgency differed slightly from the Austrian study, where a five-point scale was used for the last 4 weeks and subjects who “occasionally” had urgency were also defined as abnormal [5]. Similarly, in the Chinese community-based study, women who reported urgency “occasionally” were regarded abnormal but only in the presence of other symptoms (with criteria every 3 hour for frequency, twice per night for nocturia, and once a week or less frequent for urge incontinence) [17]. In the US study, those who reported four or more urgency episodes during the last 4 weeks and who also reported more than eight voids per day, or at least one coping strategy were classified as abnormal [4]. Some studies asked symptoms over a very long or unspecified time [3], [9], [16], [18] whereas some did not exactly describe symptom classification, questions asked, or time concerning the symptom question [7], [8], [10], [14], [15]. Overall, in all symptom-defined disorders (including overactive bladder), defining very mild/rare symptoms as pathological blurs the distinction between mild and severe, causing a considerable risk of encouraging healthy people to perceive themselves as sick [25].

In the standardization report [2], the current definition of overactive bladder includes “…usually with frequency and nocturia.”, and those symptoms are defined as complaints without any severity assessment. We defined frequency as more than 8 voids per day and nocturia as more than one void per night (as in some earlier reports [3]–[5], [18]) while those definitions are presumably clinically more relevant based on prevalences of frequency and nocturia in earlier studies [9], [16], [26], [27]. On the other hand, the definition of frequency or nocturia has no effect on the prevalence of overactive bladder when based on the current definition.

According to the standardization report [2], for the diagnosis of overactive bladder subjects with “urinary infection or other obvious pathology” should be excluded. Identification of overactive bladder without excluding known reasons causing urgency can result in overestimate of prevalence. We excluded subjects with urinary tract infection, genitourinary cancer, contracted bladder, or loop diuretics, as well as pregnant and puerperal women. In addition, we performed an analysis excluding men with benign prostatic hyperplasia as its effect on overactive bladder is unclear [1]. Some earlier studies did not report any exclusion criteria [6], [8]–[10], [14], [16]–[18], or excluded only subjects with urinary tract infection [3]. In the Austrian and Brazilian studies [5], [7], exclusions were slightly broader (for example, diabetes) than in our study and in the US study [4] even more extensive (including diabetes, congestive heart failure, and excessive fluid intake). In the Austrian study, exclusions were performed for subjects with urgency, not for the whole study sample.

Several articles have been published on the prevalence of overactive bladder (English-language MEDLINE and PubMed search to December 2006). However, many of these studies have not been population-based [5], [8], [10], [18], whereas the population-based studies [3], [4], [6], [7], [9], [14]–[17] have failed to: 1) apply the current definition of overactive bladder [3], [4], [6], [14], [15], [17], 2) report any exclusions [6], [9], [16], [17], 3) include all adult ages [3], [6], [7], [9], [15], 4) include both sexes [17], 5) report response rate or non-participants [3], [7], [9], or 6) achieve good response rate [4], [6], [9], [16] (Table 3). Furthermore, none of the earlier studies used non-response analysis to adjust for selection bias. On the other hand, as long as the symptom definition of overactive bladder is more like a description without any severity or bother assessment, there is no absolutely correct way to study the epidemiology of overactive bladder.

**Table 3:** Overview of published population-based studies assessing the prevalence of overactive bladder (OAB*) among both sexes (MEDLINE and PubMed search to December 2006) with present study (*in chronological order*)

	Origin of the study sample							
	European [3]	USA [4]	Canada [9]	Japan [6]	Porto Alegre [7]	Matsu [15]	International [16]	Finland
**Design**	Telephone interview (85%)†	Telephone interview	Telephone interview	Mailed questionnaire	Mailed (?) questionnaire	Questionnaire administered by nurse	Telephone interview	Mailed questionnaire
**Respondents**	16,776	5,204	3,249	4,570	913	1,921	19,165	3,727
**Response rate (%)**	Not reported	44.5/57.1‡	43.4	45.3	Not reported	67.0	33.0	62.4
**Age range (years)**	40–75+	18–75+	35–75+	40–100	15–55	30–79	18–70+	18–79
**Representative age-distribution of adults** §	No	Yes	No	No	No	No	Yes	Yes
**Current definition of OAB**	No	No	No	No	Yes	No	Yes	Yes
**Time period**	Not defined	Last 4 weeks	Past month	Past month	Not defined	Past 4 weeks	Not defined	Last 2 weeks
**Exclusion criteria - UTI** * **/other**	Yes/No	Yes/Yes	No/No	No/No	Yes/Yes	No/Yes	No/No	Yes/Yes
**Non-response analysis for prevalence estimate**	No	No	No	No	No	No	No	Yes
**Prevalence of OAB (%)**	17	16	18	12	19	17	12	8

* OAB, overactive bladder; UTI, urinary tract infection

† In the European study, in five out of six countries, telephone interview was used (excluding Spain where direct interviews were conducted due to lower proportion of households having telephone)

‡ Out of 11,740 participants (of 17,231 households contacted), 5,539 were considered ineligible. To calculate response rate, the number of respondents was divided by eligible participants (*the former response rate*). If same proportion of non-participants, as there were ineligible among participants (47%), were also considered ineligible, response rate was greater (*the latter response rate*).

§ Study sample was close to representative of the general population regarding age, and/or age-standardization was used.

We used postal questionnaires to assess both the prevalence of urinary symptoms and co-morbidity. Overactive bladder is a symptom-defined condition requiring self-report. Mailed questionnaires reflect urodynamics better than interview-assisted questionnaire responses [28]. Furthermore, mailed questionnaires provide more reliable information than telephone surveys in several aspects, including higher participation [29]. Telephone surveys have commonly been used, including the most cited figures [3], [4], [9], [16].

Even though most studies reported higher prevalence estimates than ours, the differences can be readily explained by dissimilarities in study procedures. For instance, Milsom and colleagues stated in their multinational study that 16.6% had overactive bladder [3]. They did not use the current definition of overactive bladder and excluded only subjects with urinary tract infection. In their study (all subjects at least 40 years old) only 54% of subjects with overactive bladder reported urgency corresponding approximately to 9.0% prevalence of urgency. Hence, based on their study population 9.0% prevalence of overactive bladder would also be overestimated due to age distribution and absence of other exclusion criteria and non-response analysis. This estimate concurs with our results.

Of the Finnish adult population, 5% are aged 80 years or more [21]. As our sample did not include this age group, we extrapolated the prevalence rates for people aged 80 years or more. Based on extrapolated prevalence rates of overactive bladder among this age group (20.0% for men and 17.5% for women), we calculated age-standardized prevelance of overactive bladder for men (6.9%) and women (9.8%). Adjustment for people aged 80 years or more did not materially change prevalence rates as they were within the confidence limits of our estimates indicating that one in twelve (8.4%) had overactive bladder in the general population. However, our study population was Caucasian, which may diminish generalizability to other ethnicities. Most reported studies also used a study population that was mainly or totally Caucasian without proper comparison of prevalence of overactive bladder between different ethnicities [3]–[5], [7], [9], [16], [18]. Consequently, there is a need to examine the effect of ethnic differences on the prevalence of overactive bladder.

Our aim was to obtain a generalizable, unbiased estimate of the prevalence of overactive bladder in both genders. Our study population from youth to old age was representative of Finnish adults in terms of socio-demographic and anthropometric factors [27], [30] and included people aged 18–79 years. Age-standardization was used to improve comparability with other studies and generalizability to other populations. Current population distribution of Finland was used so as not to underestimate prevalences. We calculated corresponding figures also using European standard population [22], but as the age structure was younger in that, the prevalence rates were slightly lower (not reported). To further improve the generalizability, we estimated corrected prevalence of overactive bladder with adjustment for people aged at least 80 years. After adjustment for people aged 80 years or more, the results remained substantially the same. A good response rate was achieved, but to further improve the validity, we estimated the corrected prevalence of overactive bladder with adjustment for selection bias due to non-response. We corrected prevalence for selection bias on the assumption that overactive bladder was equally common among non-responders and in late responders. The corrected estimate was smaller, indicating that naїve analysis overestimates prevalence.

Our results suggest that the prevalence of overactive bladder has been overestimated so that the true prevalence is approximately half of that proposed earlier. Overactive bladder affects approximately one out of twelve adults of Caucasian origin.

## Supporting Information

Table S1 Number of subjects in different analyses and in overactive bladder analysis.
(0.07 MB DOC)
