# Supplementary material for: Is the Prevalence of Overactive Bladder Overestimated? A Population-Based Study in Finland
Source: PLoS One. 2007 Feb 7;2(2):e195. doi: 10.1371/journal.pone.0000195 (PMC1805814; doi:10.1371/journal.pone.0000195)
Supplement: Table S1 — Number of subjects in different analyses and in overactive bladder analysis. (0.07 MB DOC) [file pone.0000195.s001.doc]

**Table S1.** Number of subjects in different analyses and in overactive bladder analysis by mailing round in Finland, 2003-2004

| **Age group (years)** | **Original sample** | **Eligible sample** |  | **Partici- pants** | **Urgency analysis†** | **Overactive bladder analysis†** | **Subjects by response round in overactive bladder analysis** | | | | **OAB* without BPH* analysis†** | **All OAB symptomsanalysis†** |
| --- | --- | --- | --- | --- | --- | --- | --- | --- | --- | --- | --- | --- |
|  |  |  |  |  |  |  | **1^st^** | **2^nd^** | **3^rd^** | **Missing‡** |  |  |
| **Men** |  |  |  |  |  |  |  |  |  |  |  |  |
| 18–29 | 800 | 796 |  | 388 | 386 | 385 | 167 | 121 | 82 | 15 | 385 | 379 |
| 30–39 | 800 | 798 |  | 428 | 425 | 424 | 188 | 139 | 77 | 20 | 424 | 415 |
| 40–49 | 600 | 600 |  | 335 | 330 | 324 | 155 | 96 | 59 | 14 | 316 | 314 |
| 50–59 | 300 | 300 |  | 197 | 194 | 190 | 99 | 44 | 34 | 13 | 175 | 188 |
| 60–69 | 300 | 298 |  | 226 | 221 | 208 | 103 | 57 | 21 | 27 | 160 | 199 |
| 70–79 | 200 | 196 |  | 151 | 144 | 118 | 59 | 29 | 14 | 16 | 77 | 107 |
| All men | 3,000 | 2,988 |  | 1,725 | 1,700 | 1,649 | 771 | 486 | 287 | 105 | 1,537 | 1,602 |
| **Women** |  |  |  |  |  |  |  |  |  |  |  |  |
| 18–29 | 800 | 797 |  | 510 | 504 | 455 | 273 | 110 | 63 | 9 |  | 445 |
| 30–39 | 800 | 798 |  | 495 | 493 | 452 | 255 | 117 | 66 | 14 |  | 441 |
| 40–49 | 600 | 597 |  | 408 | 404 | 395 | 233 | 97 | 44 | 21 |  | 385 |
| 50–59 | 300 | 299 |  | 213 | 210 | 199 | 108 | 55 | 22 | 14 |  | 187 |
| 60–69 | 300 | 299 |  | 237 | 231 | 209 | 107 | 53 | 25 | 24 |  | 195 |
| 70–79 | 200 | 199 |  | 139 | 128 | 108 | 33 | 39 | 14 | 22 |  | 94 |
| All women | 3,000 | 2,989 |  | 2,002 | 1,970 | 1,818 | 1,009 | 471 | 234 | 104 |  | 1,747 |
| **Both sexes** | 6,000 | 5,977 |  | 3,727 | 3,670 | 3,467 | 1,780 | 957 | 521 | 209 |  | 3,349 |

* OAB, overactive bladder; BPH, benign prostatic hyperplasia.

† Urgency analysis was performed with all participants (without exclusions) who provided answer to urgency question; Overactive bladder analysis was the main study group (for both sexes) with exclusions; OAB without BPH analysis was performed for men after further exclusion of those with benign prostatic hyperplasia; All OAB symptoms analysis was performed for both sexes with all subjects who provided answer to every urgency, urgency incontinence, frequency, and nocturia questions after same exclusions as for overactive bladder analysis.

‡ Mailing round could not be defined due to lack of date of questionnaire completion.
